# Supplementary material for: Large-scale identification of social and behavioral determinants of health from clinical notes: comparison of Latent Semantic Indexing and Generative Pretrained Transformer (GPT) models
Source: BMC Med Inform Decis Mak. 2024 Oct 10;24:296. doi: 10.1186/s12911-024-02705-x (PMC11465786; doi:10.1186/s12911-024-02705-x)
Supplement: Supplementary file 1 — Supplementary Material 1. [file 12911_2024_2705_MOESM1_ESM.pdf]

SUPPLEMENTARY MATERIAL -

Large-scale identification of social and behavioral determinants of health from clinical notes: Comparison of Latent Semantic Indexing and Generative Pretrained Transformer (GPT) models

Sujoy Roy, PhD[1], Shane Morrell[2], Lili Zhao, PhD[3], and Ramin Homayouni, PhD[1,4*]

[1]Foundational Medical Studies, Oakland University William Beaumont School of Medicine, Oakland University, Rochester, MI, USA

[2]Quire Inc., Memphis, TN, USA

[3]Biostatistics, Beaumont Research Institute, Corewell Health, Royal Oak, MI, USA

[4]Population Health & Health Equity Research, Beaumont Research Institute, Corewell Health, Royal Oak, MI, USA

Keywords:

Social determinants of health, electronic health records, machine learning, natural language processing, clinical notes

SUPPLEMENTARY FIGURES and TABLES

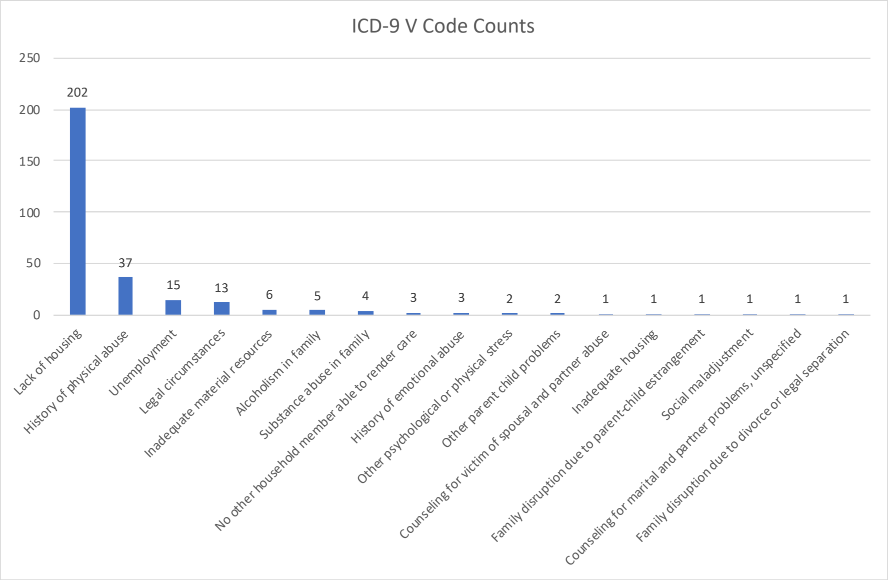

Figure S1: Distribution of patient counts with SDoH categories.

Table S1: ICD-9 codes for SBDH categories.

SBDH Category	ICD-9 Code	ICD9 Description	Keyword
Housing insecurity	V600	Lack of housing	Homeless
Physical & sexual abuse	V1541	History of physical abuse	Abused
Financial insecurity	V620	Unemployment	Unemployed
Legal circumstances	V625	Legal circumstances	Legal
Financial circumstances	V602	Inadequate material resources	Financial
Tobacco use	3051	Tobacco use disorder	Smokes
	64903	Tobacco use disorder complicating pregnancy, childbirth, or the puerperium, antepartum condition or complication	
Alcohol abuse	30500	Alcohol abuse, unspecified	Etoh
	29181	Alcohol withdrawal	
	30391	Other and unspecified alcohol dependence, continuous	
	30390	Other and unspecified alcohol dependence, unspecified	
	30501	Nondependent alcohol abuse	
	30301	Acute alcoholic intoxication in alcoholism, continuous	
	2910	Alcohol withdrawal delirium - as a primary diagnosis code,	
	5711	Acute alcoholic hepatitis - as a primary diagnosis code,	
	30300	Acute alcoholic intoxication-unspec	
	4255	Alcoholic cardiomyopathy	
	9800	Toxic effect of ethyl alcohol	
	5710	Alcoholic fatty liver	
	3575	Alcoholic polyneuropathy	
	30502	Alcohol abuse, episodic	
	30392	Other and unspecified alcohol dependence	
	5713	Alcoholic liver damage, unspec.	
	2911	Alcohol-induced persisting amnestic disorder	
	53530	Alcoholic gastritis, without mention of hemorrhage	
	2912	Alcohol-induced persisting dementia	
	E8600	Acc poisn-alcohol bevrag	
	2913	Alcohol-induced psychotic disorder with hallucinations	
	53531	Alcoholic gastritis, with hemorrhage	
	30302	Acute alcoholic intoxication.	
Cocaine abuse	30560	Cocaine abuse-unspec	Cocaine
	30561	Cocaine abuse, uncomplicated	
	30421	Cocaine dependence, continuous	
	30420	Cocaine dependence, unspecified	
	30562	Cocaine abuse-episodic	
	76075	Mat cocaine aff NB/fet.	
	30422	Cocaine dependence, episodic	
Opiate abuse	96509	Poisoning by other opiates and related narcotics	Opiate
	E8502	Accidental poisoning by other opiates and related narcotics	
	30401	Opioid type dependence, continuous	
	30550	Opioid abuse, unspecified	
	30400	Opioid type dependence, unspecified	
	30551	Opioid abuse, continuous	
	30470	Combinations of opioid type drug with any other drug dependence, unspecified	
	30471	Combinations of opioid type drug with any other drug dependence, continuous	
	30552	Opioid abuse, episodic	
	30402	Opioid type dependence, episodic	

Table S2: SBDH categories and their associated keywords (134) that were manually selected. The keywords that were chosen as the best representative of the respective SBDH categories are italicized.

**SBDH Categories (Refined Categories)**	**Keywords**
Alcohol use disorders (Alcohol abuse)	alcohol, alcoholic, drinker, ethanol, *etoh*, intoxicated, intoxication
Care provider dependency (Caregiver dependency, Mobility issues)	adl, ambulate, assistance, assisted, caregiver, carer, *caretaker*, dependent, disability, disabled, handicapped, mobility, *walker*
Compliance	*noncompliant*, non-compliant, uncooperative
Drug use disorders (Cocaine abuse, Opiate abuse)	addict, addicted, cannabis, *cocaine*, crack, drugs, ecstasy, fentanyl, heroin, illicit, ivda, lsd, marijuana, mdma, narcotics, *opiate*, opioid, overdose, pcp, snort
Education and literacy (Lack of English proficiency)	illiterate, *interpreter*, interpretor, translater, translator
Family circumstances (Physical & sexual abuse)	abuse, *abused*, abusive, violence, adopted, custody, dispute, disputes, divorce, divorced, estranged, guardian, veteran
Housing & economic circumstances (Financial circumstances, Housing insecurity, Lack of transportation)	afford, affordable, economic, evicted, eviction, finances, *financial*, hardship, *homeless*, housing, indigent, poor, retire, retired, shelter, transport, *transportation*, transported
Legal circumstances	arrest, arrested, court, illegal, imprisoned, incarcerated, incarceration, jail, jailed, *legal*, police, prison
Other psychosocial circumstances (Suicidal ideation)	behavioral, committed, counseler, counseling, counselor, delusional, delusions, homicidal, institutionalized, isolated, isolation, loneliness, mania, manic, mental, obsessive, ocd, paranoia, paranoid, psych, psychiatrist, psychologist, psychosis, ptsd, schizophrenic, suicidal, *suicide*, therapist, therapy, trauma, traumatized, ward
Tobacco use	cigar, cigarette, cigarettes, cigars, nicotine, smoker, *smokes*, smoking, tobacco
Unemployment (Financial insecurity)	*unemployed*, unemployment

Table S3: Characteristics of independently sampled gold standard (GS) patients for each SBDH category.

Variable	Alcohol abuse	Cocaine abuse	Opiate abuse	Financial circumstances
n	80	80	80	46
Male, n (%)	62 (77.5%)	52 (65.0%)	52 (65.0%)	29 (63.0%)
Age, Median (Q1, Q3)	51.0 (40.0, 60.0)	44.0 (33.5, 52.0)	47.0 (32.0, 57.5)	57.0 (42.0, 69.0)
Ethnicity, n (%)				
- ASIAN	1 (1.2%)	0 (0.0%)	3 (3.8%)	2 (4.3%)
- BLACK/AA	5 (6.2%)	6 (7.5%)	4 (5.0%)	6 (13.0%)
- HISPANIC OR LATINO	4 (5.0%)	9 (11.2%)	1 (1.2%)	3 (6.5%)
- OTHER	10 (12.5%)	13 (16.2%)	18 (22.5%)	7 (15.2%)
- WHITE	60 (75.0%)	52 (65.0%)	54 (67.5%)	28 (60.9%)
Text, n (%)	40 (50.0%)	40 (50.0%)	40 (50.0%)	23 (50.0%)
ICD-9, n (%)	40 (50.0%)	40 (50.0%)	40 (50.0%)	6 (13.0%)
True Positive, n (%)	52 (65.0%)	43 (53.8%)	36 (45.0%)	18 (39.1%)

Variable	Financial insecurity	Housing insecurity	Legal circumstances	Physical & sexual abuse
n	55	80	53	67
Male, n (%)	35 (63.6%)	63 (78.8%)	34 (64.2%)	24 (35.8%)
Age, Median (Q1, Q3)	49.5 (40.0, 59.0)	48.0 (40.0, 59.0)	60.0 (42.5, 70.0)	49.0 (34.0, 61.0)
Ethnicity, n (%)				
- ASIAN	1 (1.8%)	1 (1.2%)	1 (1.9%)	0 (0.0%)
- BLACK/AA	8 (14.5%)	12 (15.0%)	4 (7.5%)	4 (6.0%)
- HISPANIC OR LATINO	3 (5.5%)	2 (2.5%)	1 (1.9%)	4 (6.0%)
- OTHER	6 (10.9%)	12 (15.0%)	7 (13.2%)	12 (17.9%)
- WHITE	37 (67.3%)	53 (66.2%)	40 (75.5%)	47 (70.1%)
Text, n (%)	29 (52.7%)	40 (50.0%)	25 (47.2%)	27 (40.3%)
ICD-9, n (%)	15 (27.3%)	40 (50.0%)	13 (24.5%)	27 (40.3%)
True Positive, n (%)	36 (66.7%)	53 (67.1%)	26 (49.1%)	37 (55.2%)

Variable	Tobacco use
n	80
Male, n (%)	46 (57.5%)
Age, Median (Q1, Q3)	54.5 (43.8, 68.2)
Ethnicity, n (%)	
- ASIAN	1 (1.2%)
- BLACK/AA	8 (10.0%)
- HISPANIC OR LATINO	2 (2.5%)
- OTHER	7 (8.8%)
- WHITE	62 (77.5%)
Text, n (%)	40 (50.0%)
ICD-9, n (%)	40 (50.0%)
True Positive, n (%)	56 (70.0%)

SUPPLEMENTARY METHODS - DATA TRANSFORMATION

The data transformation process has been previously described by our group,[1] and is being reproduced here for completeness.

Term weighting

A term-by-patient matrix was created where the entries of the matrix were *tf-idf* weighted frequencies of terms across the patient-document collection. Weighting (normalization) blunts the effect of common terms while at the same time raising the importance of rare terms that are better discriminators between patients. Each matrix entry a_{ij} is a product of two components: term frequency (f_{ij}) and inverse document frequency (g_i):

$$g_i = -\log_2 \frac{n_i}{N} \tag{1}$$

where f_{ij} is the frequency of the ith term in the jth patient-document, n is the number of patient documents containing the term i, and N is the total number of patient documents under consideration.

Construction of the LSI model

Singular value decomposition (SVD)[2,3] was applied to the term-by-patient *tf-idf* weighted frequency matrix. Figure S2 demonstrates the process graphically.

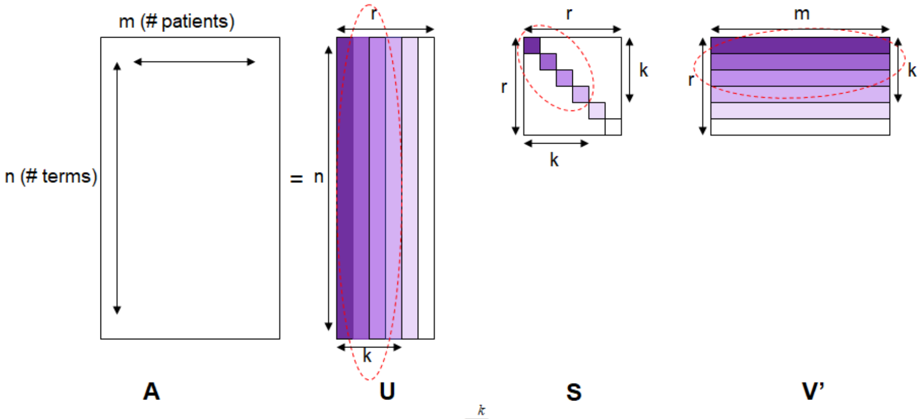

A = USV' $A_k = \sum_{i=1}^{k} A_i$ $A_i = u_i s_i v'_i$

Figure S2: Singular Value Decomposition

A data matrix A with n rows (terms) and m columns (patients) can be construed as n term row vectors in m-dimensional patient space and m patient-document column vectors in n-dimensional term space. SVD transforms the two sets of vectors into a new r-dimensional orthogonal space in which the maximum variation is expressed along the first dimension axis, as much variation independent of that is expressed along an axis orthogonal to the first, and so on. The new set of axes may reveal the true dimensionality of the data if the dataset is not inherently m-dimensional. The SVD is formulated as:

$$A = USV' \tag{2}$$

where $'$ indicates transpose of the matrix obtained by permuting the modes, i.e., transforming rows into columns and vice versa, U is $n \times r$, S is $r \times r$, and V is $m \times r$ (V' is $r \times m$). Both U and V are orthogonal, i.e., $UU' = I$ and $VV' = I$ where I is the identity matrix. S is a diagonal matrix with non-negative and non-increasing entries $\sigma_1, \sigma_2, \cdots \sigma_r$ which are known as singular values. r is the rank of the matrix, which is the number of linearly independent rows or columns of A. It is however, known from observation, for most practical datasets, $r = min(m, n)$. The third matrix V is written as a transpose so that the rows of both matrices U and V correspond to terms and patients, respectively.

The rows of A can be interpreted as term coordinates in an m-dimensional space. The axes of this space can be interpreted as rows of I (identity matrix). The SVD transforms the term coordinates to rows of U and the axes to the rows of SV'. The matrix V' acts as the rotation matrix for the original axes and the diagonal of matrix S contains the scaling factor for each axis. The U matrix can now be construed as a new transformed dataset whose rows still correspond to the original n terms but the patients are transformed into r eigen patients (factors) that are a linear combination of the original patients.

SVD is symmetric in the sense that a decomposition on the rows (terms) can be transformed into a decomposition on the columns (patients):

$$A' = VSU' \tag{3}$$

The new scaled and rotated axes and the coordinates tend to better fit the data than the original axes and coordinates. The singular values in S determine the relative importance of each axis. The first few axes capture the maximum variation in the data and the subsequent ones less so. Only the first k (where $k < r$)

factors corresponding to k largest singular values may be used to represent the data. There are two potential benefits of performing this truncation. Firstly, for large datasets (with many attributes), this translates into savings in memory space as well as analysis time, as vectors in k dimensions can be compared in less time than vectors in $min(m, n)$ dimensions. Secondly, SVD reveals the true dimensionality present in the data, where the bulk of the information content in the original m-dimensional data may be captured in a lower dimensional manifold, after axis rotation and scaling.

Calculating most significant factors

An appropriate choice for k (number of most significant factors) can be made by assessing the contribution of each of the singular values as a measure of the amount of variation captured in each dimension, and then calculating the entropy of the contributions that might be indicative of what percentage of the total number of factors may be needed. The contribution C_i of each of r singular values σs can be calculated as:

$$C_i = \frac{\sigma_i^2}{\sum_{i=1}^{r} \sigma_i^2} \tag{4}$$

and the entropy of the r contributions calculated as:

$$E = \frac{-1}{\log r} \sum_{k=1}^{r} C_k \log C_k \tag{5}$$

Entropy measures the amount of disorder in the set of variations captured in the r dimensions. The magnitude of the entropy may vary from 0 (all variation is captured in the first dimension) to 1 (all dimensions are equally important). k is calculated as $E \times r$.

Calculating association between entities

The association between any pair of entities (term-term, term-patient, patient-patient) can be calculated as the cosine of the angle between the respective k-dimensional vectors. A higher association score between a pair of entities indicates a stronger textual relationship.

Given two k-dimensional vectors:

$$A = [a_1, a_2, \cdots, a_k] \tag{6}$$

and

$$B = [b_1, b_2, \cdots, b_k] \tag{7}$$

The cosine is calculated as the normalized dot product:

$$\frac{A.B}{|A|.|B|} \tag{8}$$

which can be expanded as:

$$\frac{a_1.b_1 + a_2.b_2 + \cdots + a_k.b_k}{\sqrt{a_1^2 + a_2^2 + \cdots + a_k^2}.\sqrt{b_1^2 + b_2^2 + \cdots + b_k^2}} \tag{9}$$

MATLAB implementation

```
clear all;

% specify the stoplist file
stoplist_filename = '../data/stoplist.txt'];

% patient documents
documents_folder = '../documents/';

% TMG options
OPTIONS = struct('delimiter', 'none_delimiter', 'stoplist',
   stoplist_filename, 'stemming', 0, ...
'min_length', 2, 'min_global_freq', 2, 'local_weight', 't', ...
'global_weight', 'f', 'normalization', 'x', 'dsp', 0);

% parse documents into tf-idf weighted term-by-patient matrix A
[A, DICTIONARY, GLOBAL_WEIGHTS, NORMALIZATION_FACTORS, WORDS_PER_DOC,
   TITLES, FILES, TMG_UPDATE_STRUCT] = ...
tmg(documents_folder, OPTIONS);
```

```
% expand sparse matrix into full matrix
full_A = full(A);

% run SVD on the term by patient matrix
[U, S, V] = svd(full_A, 'econ');

% calculate the number of optimal factors
sv_list = diag(S);
sv_list_squared = sv_list.^2;
sv_squared_sum = sum(sv_list_squared);
sv_contribs = sv_list_squared./sv_squared_sum;
sv_entropy = (-1./log2(length(sv_list)) .* (sum(sv_contribs.*log2(
    sv_contribs)));
factors_final = min(ceil(sv_entropy.*(length(sv_list))), length(sv_list));

% get the truncated U and V matrices
truncated_patients_by_concepts_matrix = normr(V(:,1:factors_final)*sqrt(S
    (1:factors_final,1:factors_final)));
truncated_terms_by_concepts_matrix = normr(U(:,1:factors_final)*sqrt(S(1:
    factors_final,1:factors_final)));
```

GPT SBDH Functions

```
housing insecurity
{
        "name": "identify_housing_insecurity",
        "parameters": {
                "type": "object",
                "properties": {
                        "housing_insecurity": {
                                "type": "string",
```

```
                                "enum": ["Yes", "No"]
                        }
                }
        },
        "required": ["housing_insecurity"]
}

financial insecurity
{
        "name": "identify_unemployed",
        "parameters": {
                "type": "object",
                "properties": {
                        "unemployed": {
                                "type": "string",
                                "enum": ["Yes", "No"]
                        }
                }
        },
        "required": ["unemployed"]
}

financial circumstances
{
        "name": "identify_reported_financial_circumstances",
        "parameters": {
                "type": "object",
                "properties": {
                        "reported_financial_circumstances": {
                                "type": "string",
                                "enum": ["Yes", "No"]
```

```
                        }
                }
        },
        "required": ["reported_financial_circumstances"]
}

physical or sexual abuse
{
        "name": "identify_history_of_physical_or_sexual_abuse",
        "parameters": {
                "type": "object",
                "properties": {
                        "history_of_physical_or_sexual_abuse": {
                                "type": "string",
                                "enum": ["Yes", "No"]
                        }
                }
        },
        "required": ["history_of_physical_or_sexual_abuse"]
}

tobacco use
{
        "name": "identify_tobacco_use",
        "parameters": {
                "type": "object",
                "properties": {
                        "tobacco_use": {
                                "type": "string",
                                "enum": ["Yes", "No"]
                        }
```

```
                }
        },
        "required": ["tobacco_use"]
}

alcohol abuse
{
        "name": "identify_alcohol_abuse",
        "parameters": {
                "type": "object",
                "properties": {
                        "alcohol_abuse": {
                                "type": "string",
                                "enum": ["Yes", "No"]
                        }
                }
        },
        "required": ["alcohol_abuse"]
}

opiate abuse
{
        "name": "identify_opiate_abuse",
        "parameters": {
                "type": "object",
                "properties": {
                        "opiate_abuse": {
                                "type": "string",
                                "enum": ["Yes", "No"]
                        }
                }
```

```
        },
        "required": ["opiate_abuse"]
}

cocaine abuse
{
        "name": "identify_cocaine_abuse",
        "parameters": {
                "type": "object",
                "properties": {
                        "cocaine_abuse": {
                                "type": "string",
                                "enum": ["Yes", "No"]
                        }
                }
        },
        "required": ["cocaine_abuse"]
}

legal circumstances
{
        "name": "identify_reported_legal_circumstances",
        "parameters": {
                "type": "object",
                "properties": {
                        "reported_legal_circumstances": {
                                "type": "string",
                                "enum": ["Yes", "No"]
                        }
                }
        },
```

```
        "required": ["reported_legal_circumstances"]
}
```


REFERENCES

[1] Roy, S., Curry, B.C., Madahian, B., et al. Prioritization, clustering and functional annotation of micrornas using latent semantic indexing of medline abstracts. In *BMC bioinformatics*, volume 17, pages 131–142. BioMed Central, 2016.

[2] Golub, G.H. and Van Loan, C.F. *Matrix computations*, volume 3. JHU Press, 2012.

[3] Skillicorn, D. *Understanding complex datasets: data mining with matrix decompositions*. CRC press, 2007.
