# Supplementary material for: Large-scale identification of social and behavioral determinants of health from clinical notes: comparison of Latent Semantic Indexing and Generative Pretrained Transformer (GPT) models
Source: BMC Med Inform Decis Mak. 2024 Oct 10;24:296. doi: 10.1186/s12911-024-02705-x (PMC11465786; doi:10.1186/s12911-024-02705-x)

Magnified heatmaps for SBDH categories

Tobacco use

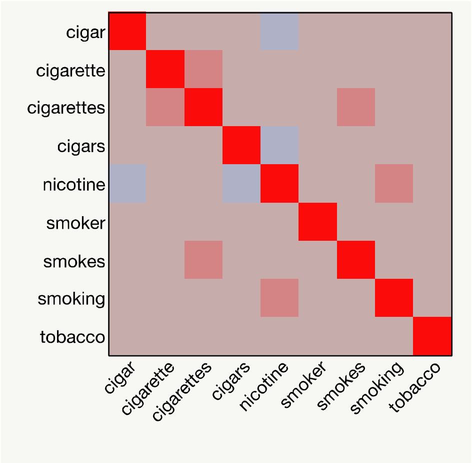

Alcohol abuse

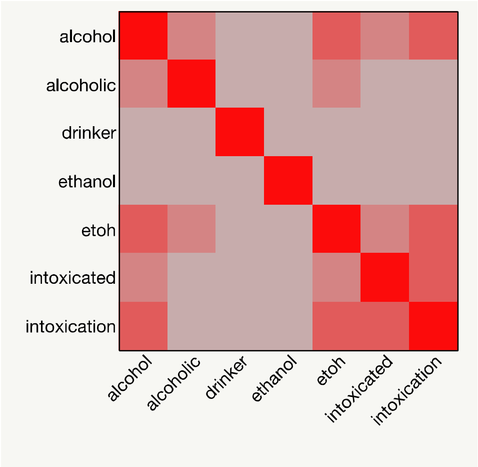

Drug abuse

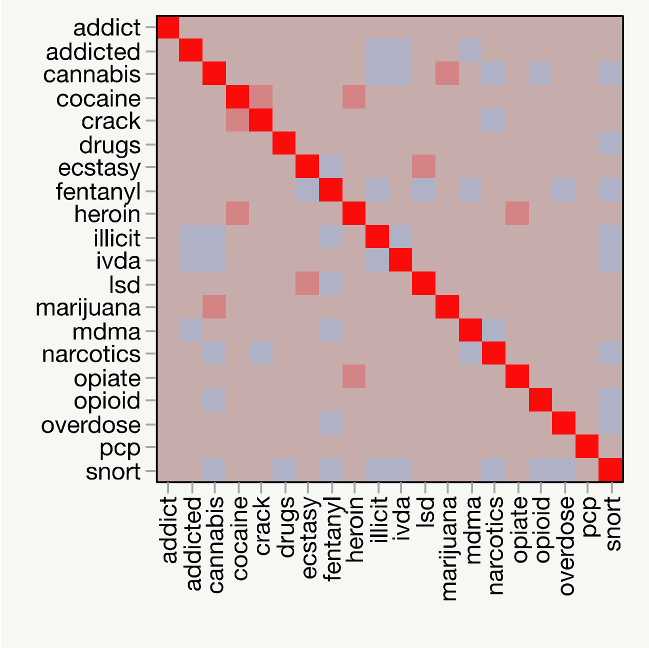

Mobility and transportation

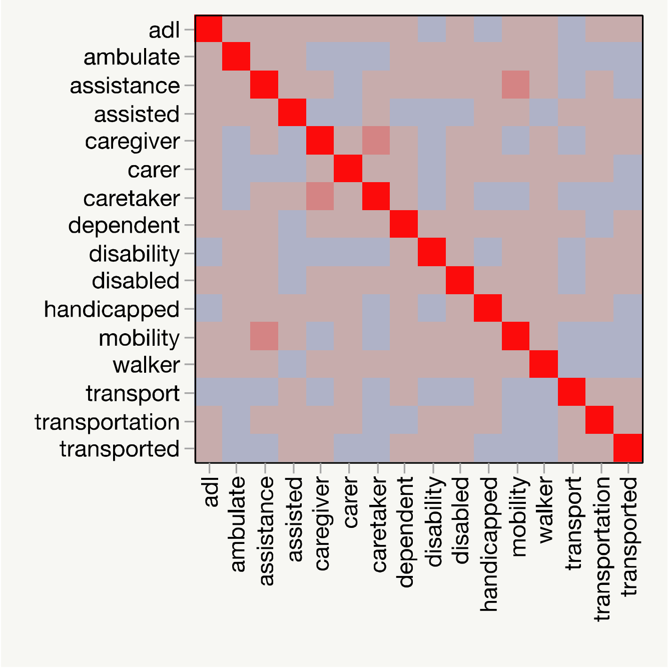

Other psychosocial circumstances

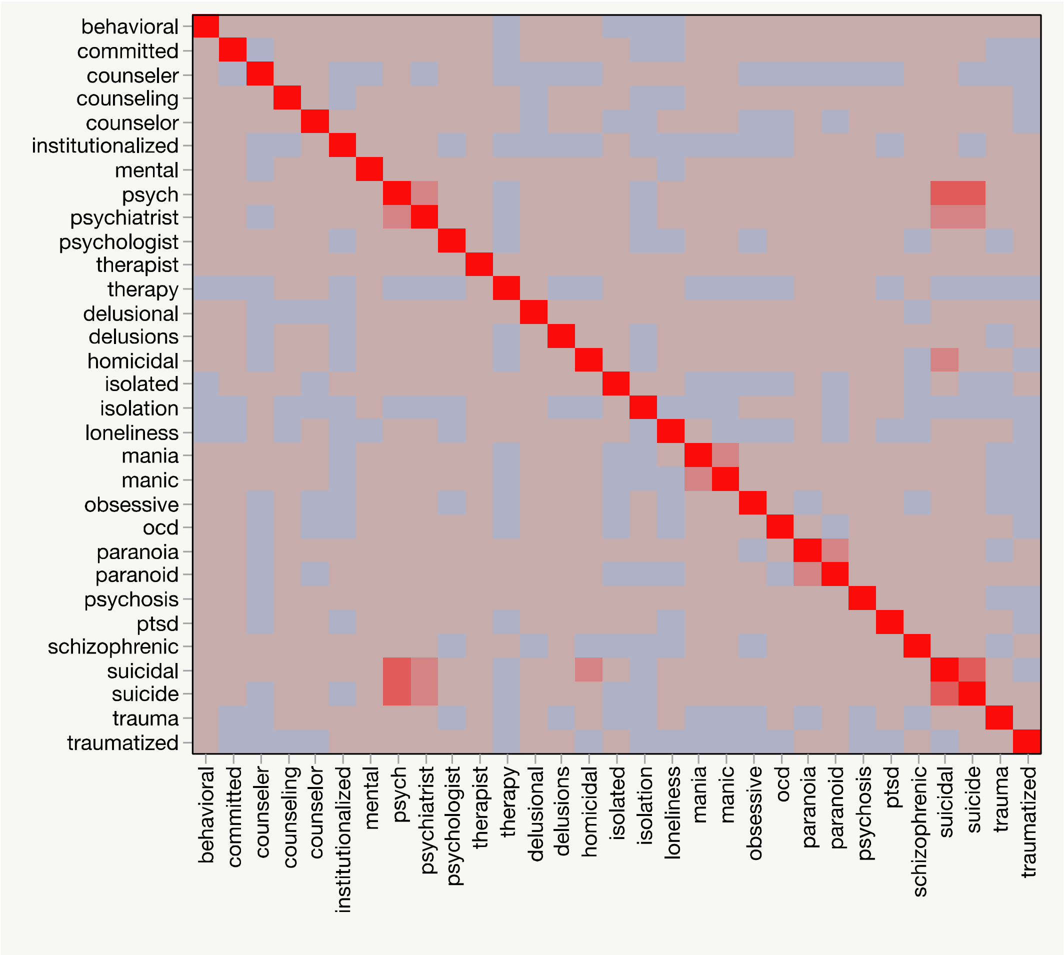

Family circumstances

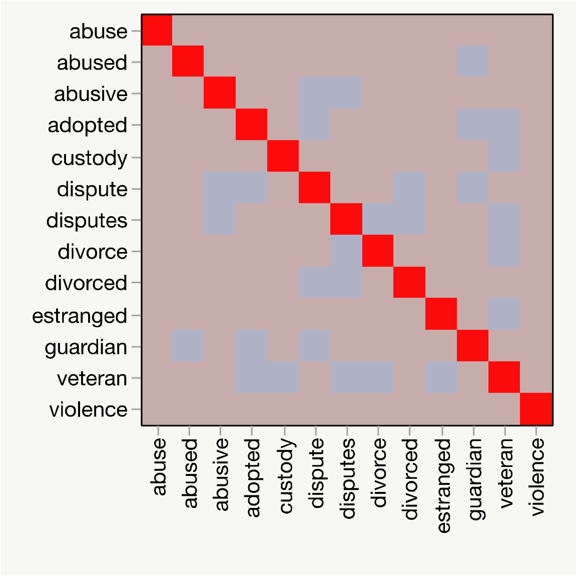

Housing and economic circumstances

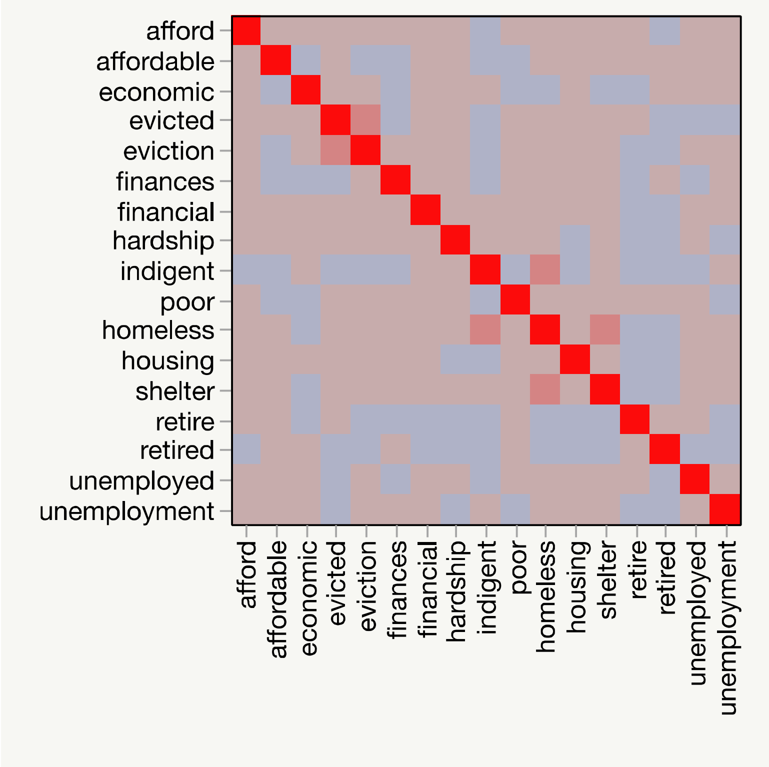

Education and literacy

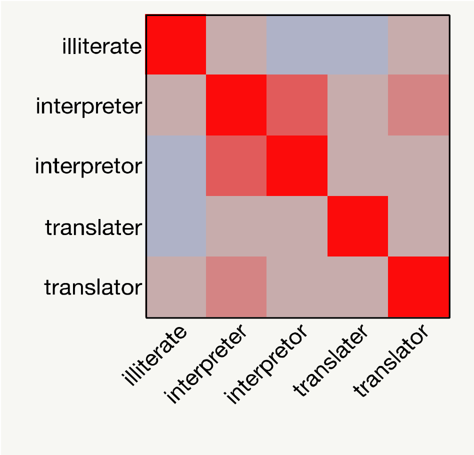

Legal circumstances

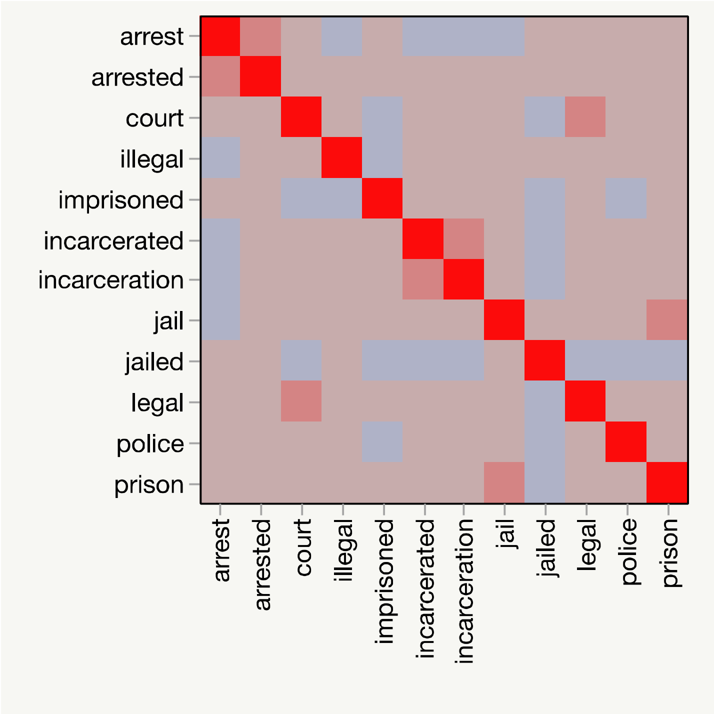

Compliance

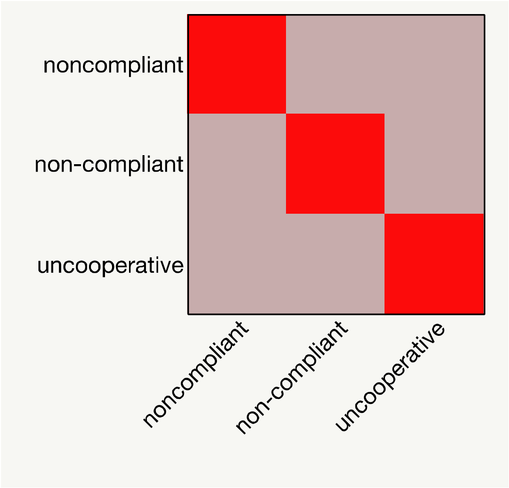

Supplement: Supplementary file 2 — Supplementary Material 2. [file 12911_2024_2705_MOESM2_ESM.pdf]
